# The Effects of Energy Drinks on the Cardiovascular System: A Systematic Review

**DOI:** 10.1007/s11886-025-02293-w

**Published:** 2025-11-14

**Authors:** Joseph Mandato, Rei Kola, Troy Tyson, Luke Laffin, Robert Bales

**Affiliations:** 1 Cleveland Clinic Akron General Family Medicine Residency, Akron, OH 44307 USA; 2 MetroHealth Family Medicine Residency, Cleveland, OH 44195 USA; 3 Ohio University Heritage College of Osteopathic Medicine-Cleveland, Cleveland, OH 44195 USA; 4 Cleveland Clinic South Pointe Hospital, 20000 Harvard Ave, Warrensville Heights, OH 44122 USA; 5 Cleveland Clinic South Pointe Hospital Medicine, Warrensville, OH 44122 USA; 6 Cleveland Clinic Main, Campus-Cardiovascular Medicine: 9500 Euclid Avenue, Cleveland, OH 44195 USA

**Keywords:** Blood pressure, ECG, Heart rate (Variability), Caffeine

## Abstract

**Introduction:**

Energy drinks are widely marketed to enhance alertness and performance, making them a rapidly growing industry valued at $73.99 billion. These beverages typically contain caffeine and other stimulants, but their consumption has raised concerns about potential cardiovascular risks, including arrhythmias, tachycardia, and hypertension, particularly among young adults. While moderate caffeine intake may offer health benefits, excessive consumption of energy drinks has been linked to increased cardiovascular risks. This systematic review aims to explore the cardiovascular effects of energy drink consumption, identify knowledge gaps, and guide clinicians in making recommendations.

**Methods:**

A comprehensive literature search was conducted across multiple databases, including CENTRAL, CINAHL, MEDLINE, PubMed, and others. Studies were screened for those involving energy drink consumption and cardiovascular outcomes such as heart rate, blood pressure, and ECG changes. After screening abstracts, full-text articles were reviewed based on inclusion criteria: participants aged 13+, energy drink consumption, and relevant cardiovascular endpoints.

**Results:**

A total of 1,444 references were screened, resulting in 37 studies with 1,597 participants (mean age 22.6). *Red Bull* and *Monster* were the most studied brands. Results showed significant cardiovascular effects, including increased heart rate (60.9%), systolic blood pressure (53.8%), diastolic blood pressure (61.5%), and QTc interval prolongation (63.2%). Other ECG changes, such as PR interval and T-wave alterations, were observed in 57.9% of studies.

**Conclusion:**

This review highlights the cardiovascular risks of energy drinks, including increased heart rate, blood pressure, and QTc prolongation. Future research should focus on at-risk groups and long-term effects.

**Supplementary Information:**

The online version contains supplementary material available at 10.1007/s11886-025-02293-w.

## Introduction

Energy drinks are a category of beverages which claim to increase energy, alertness, and physical performance by providing caffeine or caffeine-substitutes. Guarana, sugars, taurine, ginseng, B vitamins, glucuronolactone, yohimbe, carnitine, and bitter orange have all been found as are additional ingredients in energy drinks​ [1]​. The current size of the energy drink market is 73.99 billion dollars globally. Additionally, this market grew 9% from 2021 to 2024 [2]​ The most popular energy drink company, *Red Bull*, recorded 7.29 billion dollars in total sales in the year 2023​ [3]​. Other companies that heavily market these high caffeine drinks include *Monster*,* Reign*, and *C4*.

Most energy drinks have standard ingredients including water, caffeine, electrolytes, and B vitamins ​ [5]​. The other ingredients in energy drinks may have an impact on the cardiovascular system as-well such as guarana, taurine, L-carnitine, and vitamin B3. Guarana is a plant material used in energy drinks which contains more caffeine than coffee beans ​ [6]​. Taurine acts as a B-amino acid that regulates calcium transport and homeostasis, regulates myofibril sensitivity to calcium, increased nitric acid availability, and inhibiting the renin-angiotensin-aldosterone system. One review found reductions in HR, SBP, DBP, and NYHA classification with taurine supplementation ​ [7]​. L-carnitine facilitated long-chain fatty acid movement into the mitochondria. L-carnitine supplementation improves performance and exercise metabolism at risk of increasing TMAO, a pro-anthrogenic factor​ [8]​. Niacin supplementation historically was used to lower low-density lipoprotein cholesterol, but studies do not show a benefit in lowering lipoprotein(a)​ [9]. Terminal metabolites of niacin even increase CVD risk. Further studies are needed to show how high doses of all these compounds in combination affect cardiovascular disease risk.

The most potent ingredient, caffeine, is the substance that most comes into question when given in high amounts. The effect severity is primarily influenced by factors such as preexisting health conditions, the amount of caffeine consumed, and the duration of exposure. Although caffeine, either alone or with other substances, can interact with prescription and over-the-counter medications, studies show that typical caffeine doses generally cause only minor changes in heart rate, blood pressure, sympathetic activity, and cardiac electrophysiological function ​ [10]. Caffeine toxicity includes tachycardia, anxiety, agitation, restlessness, insomnia gastrointestinal disturbances, and tremors​ [11] Caffeine affects adenosine receptors, phosphodiesterase, and ryanodine receptors based on dose [12]. Habitual consumption of moderate amounts of caffeine, defined as 2–3 cups of coffee daily, has not been shown to trigger arrhythmogenesis [13]. Excessive use is linked to PVCs, ventricular fibrillation, atrial fibrillation, and myocardial infarction​^11^​. Energy drinks possess also higher caffeine content and concentration making overdose more likely [14]. Some individuals who consume caffeine go on to develop dependence, making energy drinks a cause for concern. Caffeine abuse is defined as the uncontrollable urge to drink caffeine, and tolerance and abstinence mechanisms underscore dependence [11]. Caffeine withdrawal symptoms, mediated through this increased number of adenosine receptors, include headache, fatigue, drowsiness, depressed mood, difficulty concentrating, and flu-like symptoms. These symptoms can be reduced by using coffee with lower caffeine concentrations [20]​. It is important to differentiate regular coffee and tea use from energy drinks due to their high caffeine content, as energy drinks have upwards of 200-300 mg of caffeine a serving, containing more caffeine than 2–3 cups of coffee.

Energy drinks are also now being used for performance and have the potential to be abused in high amounts. Additionally, increased youth targeted marketing of an addictive substance could lead to dependence at a very young age and for those at risk of abuse. Another cause for concern, specifically in the adolescent population, is the identification of correlations between energy drink consumption and various factors such as BMI, poor academic performance, physical inactivity, and unhealthy dietary habits [21]. Caffeine abuse, dependence, and withdrawal can lead to excessive coffee and energy drink consumption. Energy drink marketing uses flavor profiles that target younger individuals. The highest prevalence of energy drink consumers are adolescents and young adults [22]. Energy drink use associates positively with nicotine, alcohol, binge drinking, and cannabis use [23]. Research is important to prevent acute and chronic substance-abuse problems in adolescents and young adults. Therefore, our aim is to conduct a systematic review on the cardiovascular effects of energy drink consumption with further aims to elucidate the risks, identify gaps of knowledge, and empower primary care providers to recommend safer energy drink consumption.

## Methods

A literature search was conducted with the assistance of a research librarian on cardiovascular effects on energy drink consumption. Covidence (NIH, Bethesda, MD) was used to extract relevant articles for the study. Databases searched included CENTRAL, CINAHL, Citation searching, ClinicalTrials.gov, Embase, Google Scholar, MEDLINE, PsycINFO, PubMed, Scopus, Web of Science, and World Health Organization (WHO). References were then pulled into Covidence (NIH, Bethesda, MD) and the final databases used for collection were Embase, MEDLINE, and Web of Science. Abstracts were screened by 2 reviewers, assessing if the study contained Energy drinks and cardiovascular outcomes. For conflicts, a third reviewer reviewed the conflicts and made the final consensus on if the study moves through to full text review. The full text review was then conducted and screened based on our inclusion and exclusion criteria.

This criterion is as follows:

*Inclusion Criteria*:

1. Participants aged 13 or older.
2. Energy drink consumption (brand name or synthetic with caffeine).
3. Must contain cardiovascular outcomes and endpoints including but not limited to heart rate, blood pressure, cardiac output, ECG findings, doppler imaging, etc.

*Exclusion Criteria*:

1. Study containing caffeine only in absence of energy drinks
2. Pregnancy or breastfeeding.
3. Full texts could not be obtained.
4. Case studies.
5. Study obtained the wrong endpoints set by the authors.
6. Letters to editor.
7. Systematic or metanalysis reviews.
8. Deemed irrelevant to topic by authors.
9. Animal study designs.
10. Studies not obtainable in English.
11. Poor or non-scientific research protocol.
12. Article was an editorial.
13. Duplicate studies.
14. Book chapters articles.
15. Poster presentations.

For resulting conflicts, peer-to-peer review and decision was conducted to get final full text articles for extraction. PRISMA further outlining the review process is present in Fig. 1 (Appendix A). The authors then divided the final full texts, using our extraction protocol in the figure below, with each getting 19 and 18 articles respectively. A final consensus was then reached via peer to peer. This contained a thorough review of the data and conflicting reviews. The Systematic Review criteria can be seen in Table 1 (Appendix A).

## Results

1444 references were imported for screening, and initially 7 duplicates were identified manually and 190 duplicates identified by Covidence. 1247 studies were screened against title and abstract resulting in 1020 studies being further excluded. 227 studies were then assessed for full-text eligibility, and 190 studies excluded. 34 studies’ full texts could not be obtained despite the authors best efforts, 31 were case studies, 29 contained the wrong endpoints set by the authors, 23 were Letters to Editor, 20 were already completed systematic or Meta-analysis reviews, 17 were further deemed irrelevant to topic, 8 were animal study designs, 6 were not obtainable in English, 6 were further excluded due to research protocol, 5 studies were editorials 4 studies were further found to be duplicates, 3 studies contained Caffeine-only, 2 were not published works but book chapters, and 2 studies were poster presentations. This left 37 studies to be systematically reviewed.

Quality assessments of the data were conducted by both the authors. The authors determined that 13 studies were of high quality, 10 were of low quality, and 14 did not have enough evidence to grade appropriately for discussing allocation of concealment, For blinding of participants and personnel, the authors determined that 16 studies were of high quality, 15 were of low quality, and 6 did not have enough evidence to grade appropriately. The authors determined that 10 studies were of high quality, 17 were of low quality, and 10 did not have enough evidence to grade appropriately for blinding of outcome assessment. For the assessment of incomplete outcome data, the authors determined that 25 studies were of high quality, 6 were of low quality, and 6 did not have enough evidence to grade appropriately. Lastly, for selective reporting, the authors determined that 6 studies were of high quality, 14 were of low quality, and 17 did not have enough evidence to grade appropriately.

Of the 37 studies that were reviewed, 10 studies located in the United States of American, 3 studies were in the Middle East, 19 studies were in Europe, 3 studies were in Eastern Asia, and 1 study was conducted in South America. The study designs included 17 randomized control trials, 8 nonrandomized experimental studies, and 12 cross sectional study designs. Of those 37 studies, there were a total of 1597 participants, for a mean of 43.2 participants per study. All studies reported age, with the mean age equating to 22.6 years old (*n* = 35). Race was extracted for the studies, but only 4 studies reported varying races, with those 4 studies being within the United States. Of the studies that reported biological sex (*n* = 1420 people), 770 (54.2%) were male and 650 (45.8%) were females (*n* = 32). Of the studies that reported BMI averages (*n* = 819 people), the mean BMI of 22.7 kg/m2 was reported (*n* = 32). For the United States alone, studies reported BMI (139 people) and was found to have an average BMI of 24 kg/m2 (*n* = 4).

All 37 study designs used a form of energy drink that contained caffeine. 15 studies included *RedBull*, 4 included *Monster*, 1 included *Celcius*, 1 Included *5-hour Energy*, 2 included *C4*, and 14 studies did not specify the energy drink used. Study designs used vary levels of caffeine, from low (Caffeine < 150 mg) to higher dosages (Caffeine > 150 mg) of caffeine. Of the studies that specified caffeine dosage, the average dose of caffeine per study was 147.6 mg (*n* = 17). Studies that included higher dosages of caffeine (*n* = 16), the average dose of caffeine per study was 224.6 mg. For inclusion of exercise into the study protocol, 7 studies (19%) included an exercise protocol. It is also worth noting that reported patient symptoms were extracted during the systematic review. 7 Studies (19%) also included outcomes based on patients who reported symptoms after consuming energy drinks.

Various hemodynamic parameters were extracted for the study. For extraction, studies were evaluated based on a statistically significant increase (P-Value ≤ 0.05) for that hemodynamic parameter. 14 studies (60.9%) showed statistical increase of the participants heart rate (*n* = 23). 3 studies showed a statistical increase in heart rate variability (*n* = 23). 15 studies (53.8%) of studies showed a statistical increase in systolic blood pressure (*n* = 28). 16 studies (61.5%) of them showed a statistical increase in Diastolic blood pressure (*n* = 26). 4 studies (40%) show a statistically significant increase in mean arterial pressure (*n* = 10). 2 Studies (33%) showed a statistical increase in cardiac output. 1 study (33%) showed a statistical increase in stroke volume.

EKG measurements were also taken in these studies containing energy drinks. Out of the 37 total studies, 19 of them (51.4%) had EKG measurements and outcomes embedded in the study design. 12 of these studies (63.2%) investigated QTc changes (*n* = 19). Out of these 12 studies, 8 studies (66.7%) showed statistically significant lengthening of the QTc (P-Value ≤ 0.05). Also, 11 (57.9%) studies contained other EKG changes included but not limited to changes in PR interval, QRS, and T wave changes (*n* = 19).

## Discussion

### Study Quality

The quality of the studies included in this review varied significantly. While 25 studies were rated high quality in some respects, such as handling incomplete outcome data and ensuring proper allocation concealment, notable Limitations were observed in other areas, including blinding and selective reporting. Of the 16 studies that rated highly for blinding of participants and personnel, 15 studies were rated low quality due to potential biases in outcome assessments. The concern about selective reporting—where only certain outcomes or data are reported, often influenced by the study’s hypothesis or sponsor—was a recurring issue in 14 studies. This could potentially skew findings, particularly in studies investigating subtle and complex cardiovascular changes fluctuations in blood pressure. Consequently, the variations in study quality, especially in the less rigorously designed studies, raise concerns about the overall validity of some of the conclusions regarding the risks associated with energy drink consumption.

Moreover, a significant gap across the studies was the representation of at-risk populations, such as individuals with pre-existing cardiovascular conditions or those with other health conditions like obesity, diabetes, or hypertension. Most studies predominantly focused on young, healthy participants, typically those who were caffeine-naive, thus limiting the ability to extrapolate findings to individuals who may be more vulnerable to the cardiovascular effects of energy drinks. Therefore, the results might not reflect the potential risks that individuals with underlying health conditions or those consuming caffeine regularly might face when exposed to the stimulatory effects of energy drinks.

### Study Design

The review included a combination of randomized controlled trials (RCTs), cross-sectional studies, and experimental studies, with RCTs providing stronger evidence of causal relationships. However, nonrandomized studies and cross-sectional studies often lacked sufficient control for confounding factors, limiting the reliability of their conclusions. Furthermore, many studies included only short-term observations, which restricts our understanding of the long-term cardiovascular effects of energy drink consumption, particularly in individuals with chronic health issues. For example, some studies suggest that energy drink consumption acutely increases heart rate and blood pressure [17, 24]. This results in the long-term implications of these transient changes remaining poorly understood.

An important limitation of the existing body of research is that studies overwhelmingly focus on healthy individuals, frequently caffeine-naive, without addressing how these drinks might affect those with existing cardiovascular conditions. For instance, several studies have shown that energy drinks, especially those with high caffeine content, can cause significant increases in systolic and diastolic blood pressure​ [24]. This can be particularly concerning for individuals with hypertension. Additionally, the studies included did not assess how chronic consumption might interact with other risk factors such as obesity, age, or family history of cardiovascular diseases. Studies often used doses for their study, such as one *Redbull*, which do not reflect the frequency of some individuals in the United States.

### Cardiovascular Effects

Energy drinks have been associated with a prolongation of the QTc interval, a well-established marker of potential arrhythmic risk. In example, A study from Shah and colleagues showed a 10ms increase in QTc compared to the placebo up to 4 h after consumption [24]. These findings could suggest a potential risk for fatal arrhythmias especially when ingesting higher than maximum dosages of energy drinks, particularly torsade’s de pointes. This is particularly concerning for individuals with pre-existing arrhythmias or those on medications that prolong the QT interval.

In addition to QTc prolongation, studies have also documented increased heart rate and elevated blood pressure following energy drink consumption, with some studies noting that systolic blood pressure could increase by as much as 4 mm Hg and diastolic by 6 mm Hg, which may exert additional strain on the cardiovascular system​ [26, 27]. Acute increases in afterload due to energy drink’s effect on blood pressure may affect vulnerable populations, and excessive and chronic consumptions may increase the risk of chronic hypertension and structural heart disease. These findings align with studies that highlight the need for caution regarding energy drink consumption in populations with a history of arrhythmias or those with high risk, such as those with congenital QTc prolongation.

Interestingly, there are conflicting findings on the impact of energy drinks on QTc prolongation, with some studies failing to show any significant changes to QTc following acute consumption [28, 29]. These discrepancies could be attributed to variations in the type of energy drink used, study design, or the specific health status of participants. However, the risk remains concerning enough to warrant further research, particularly regarding the effects of energy drinks on individuals with pre-existing cardiovascular conditions.

Energy drinks were also shown to influence other electrocardiographic parameters, including the QRS interval, T-wave morphology, and atrial conduction times. A study found a significant increase in the QRS interval length (+ 2.67 ms, *p* = 0.03), indicating potential alterations in ventricular conduction [30]. Additionally, T-wave changes were observed in 64.3% of subjects, with some participants experiencing flattened or inverted T-waves, and one subject displaying ST depression [31]. These changes may indicate altered repolarization, which could contribute to arrhythmias. Notably, PR interval duration decreased over time (1.93 ms at 1 h and 4.1 ms at 2 h), suggesting possible effects on atrial conduction [32]. Furthermore, supra-ventricular extra-systoles and a decrease in the RR interval were also reported, highlighting the broader impact on the cardiac cycle [33, 34]. These findings suggest that while energy drinks can alter several cardiovascular parameters, the clinical significance of these changes, particularly in the context of arrhythmogenesis, warrants further investigation. However, one RCT study did show no significant differences were noted in other measures such as PR interval, QT interval, or heart rate, making these conclusions unclear [28].

While the increase in blood pressure and heart rate following energy drink consumption is generally well-established, studies often**”** underscored the potential long-term cardiovascular strain posed by energy drinks [35, 36]. This is especially concerning individuals who may consume them regularly, whether as a stimulant for work, exercise, or other daily activities. Additionally, certain ingredients, such as taurine and guarana, may have synergistic effects with caffeine, potentiating these hemodynamic changes [37, 38].

It is important to note that some Literature does support daily caffeine use in moderation has rather positive effects. In one study, researchers analyzed data from 382,535 individuals without a history of heart disease to determine if coffee consumption influenced the development of heart disease or stroke over a 10-year follow-up period. The participants had an average age of 57, and half were women. Overall, drinking two to three cups of coffee daily was Linked to the greatest benefits, reducing the risk of coronary heart disease, heart failure, arrhythmias, or death from any cause by 10%−15%. The lowest risk of stroke or heart-related death was observed in those who consumed one cup of coffee per day ​^15^​.This showed the optimal benefit occurring in those who drank two to three cups daily, while consuming more or fewer cups provided less benefit.

### Participant Population Discrepancies

A particularly crucial aspect of this review is the lack of data on individuals who are at increased cardiovascular risk, such as those with hypertension, arrhythmias, or metabolic disorders. While young, healthy adults are frequently the focus of energy drink research, individuals in higher-risk groups may experience more severe cardiovascular effects due to underlying health conditions. Some people cannot tolerate a transient increase in afterload or reduction of parasympathetic response due to caffeine’s increase in catecholamines and action on the phosphodiesterase. For instance, studies have demonstrated that overweight or obese individuals may have an exaggerated cardiovascular response to energy drink consumption, such as increased blood pressure and reduced parasympathetic activity [39]. This heightened response could make them more vulnerable to negative cardiovascular outcomes, including arrhythmias and hypertension.

Additionally, children and adolescents may be at particular risk, as energy drink consumption has been linked to increased systolic and diastolic blood pressure and altered heart rhythms, potentially exacerbating developmental cardiovascular issues [33]. Given the growing popularity of energy drinks among youth, the long-term implications for this demographic, particularly those with existing heart rhythm conditions, warrant further investigation. One specific study points out that energy drinks might impair autonomic function in overweight and obese individuals, potentially increasing the risk of arrhythmias [36].

Recent studies have highlighted that gender differences may also play a role in how energy drinks affect cardiovascular parameters. For example, a study by Monnard and colleagues found that women might experience a greater reduction in cerebral blood flow velocity (CBFV) after consuming energy drinks compared to men, suggesting that energy drinks may have a more significant cardiovascular impact in women [40]. This could be due to differences in cardiovascular physiology between genders or the hormonal influences that may alter how the body responds to stimulants like caffeine. Understanding these potential gender-specific effects is crucial for refining public health recommendations and guiding more personalized interventions for energy drink consumers.

## Conclusion

The findings from this review indicate that while energy drinks are commonly consumed as a quick energy boost or cognitive enhancer, their cardiovascular effects—particularly in relation to blood pressure, heart rate, and QTc interval prolongation—raise significant concerns. Acute increases in blood pressure and heart rate, along with QTc prolongation, particularly in vulnerable populations, underscore the potential risks of energy drink consumption. Especially when individuals can buy and consume as many energy drinks as they want from the store. For individuals with pre-existing cardiovascular conditions or those predisposed to arrhythmia, the use of energy drinks could increase the risk of adverse outcomes such as arrhythmias or heart failure. The need for targeted health guidance and risk stratification for energy drink consumption, especially for high-risk groups, is evident.

Future research should focus on elucidating the effects of energy drinks on at-risk populations, such as those with hypertension, obesity, and arrhythmias. Additionally, more research is needed into how chronic energy drink consumption might interact with long-term health conditions, particularly concerning cardiovascular and metabolic health. A researcher may be interested in prospective, case-control trials investigating common chronic diseases. This research is needed to explore the long-term cardiovascular effects of energy drink consumption in individuals with various risk factors, such as age, obesity, hypertension, and existing heart conditions. Studies focusing on energy drink consumption in women, children, and older adult populations are also warranted to better understand how these groups may differ in their cardiovascular response. Finally, multi-center, global studies will be critical to understand the wide-reaching implications of energy drink consumption, especially in areas where energy drink consumption patterns are less well-studied.

## Study Limitations

While this review provides valuable insights into the cardiovascular effects of energy drinks, several limitations must be acknowledged. First, most studies focused on healthy, young adults who were often caffeine-naive, limiting the generalizability of the findings to other demographic groups. The exclusion of individuals with pre-existing cardiovascular conditions or other health issues means that the risks to these vulnerable populations remain underexplored. Energy drinks may have different effects on individuals with conditions such as hypertension, arrhythmia, or metabolic disorders, and future studies should address these gaps. Also, variability in study design and quality poses a limitation. While randomized controlled trials (RCTs) offer stronger evidence of causal relationships, many studies were not randomized or lacked sufficient controls for confounding factors, such as baseline health conditions or lifestyle variables. This lack of rigorous control diminishes the reliability of some conclusions, especially when studies fail to account for other factors like diet, exercise, or medication use. Moreover, many studies only assessed the acute effects of energy drinks, leaving the long-term cardiovascular consequences of habitual consumption largely unexplored. Additionally, more research is needed into the effects of energy drink consumption in the adolescent population, as literature suggests that consumption in this patient population is correlated with various health concerns as well as academic and behavioral issues. This could indicate a need for future systematic review of the literature surrounding energy drink consumption in this patient population.

## Supplementary Information

Below is the link to the electronic supplementary material.

Supplementary Material 1 (PDF 198 KB) 

## Data Availability

No datasets were generated or analysed during the current study.

## Key References

1. Aonso-Diego G, Krotter A, García‐Pérez Á. Prevalence of energy drink consumption world‐wide: A systematic review and meta‐analysis.*Addiction*. 2024;119(3):438–463. doi:10.1111/add.16390.

- The systematic review and meta-analysis synthesizes global data on energy drink consumption, providing prevalence of use estimates across various populations. This work offers a comprehensive benchmark for understanding consumption patterns and guiding our research on its health impacts.

2. Banks NF, Rogers EM, Helwig NJ, et al. Acute effects of commercial energy drink consumption on exercise performance and cardiovascular safety: a randomized, double-blind, placebo-controlled, crossover trial.*J Int Soc Sports Nutr*. 2024;21(1):2297988. doi:10.1080/15502783.2023.2297988.

- This research conducted a randomized, double-blind, placebo-controlled crossover trial to evaluate the short-term impact of commercial energy drinks on exercise performance and cardiovascular safety. Their findings provided evidence on acute safety considerations relevant to athletic and general populations.

## Declarations

We adhere to the preprint policy as requested.
