# Supplementary material for: The Effects of Energy Drinks on the Cardiovascular System: A Systematic Review
Source: Curr Cardiol Rep. 2025 Nov 14;27(1):156. doi: 10.1007/s11886-025-02293-w (PMC12618331; doi:10.1007/s11886-025-02293-w)
Supplement: Supplementary file 1 — Supplementary Material 1 (PDF 198 KB) [file 11886_2025_2293_MOESM1_ESM.pdf]

Appendix A: Tables and Figures

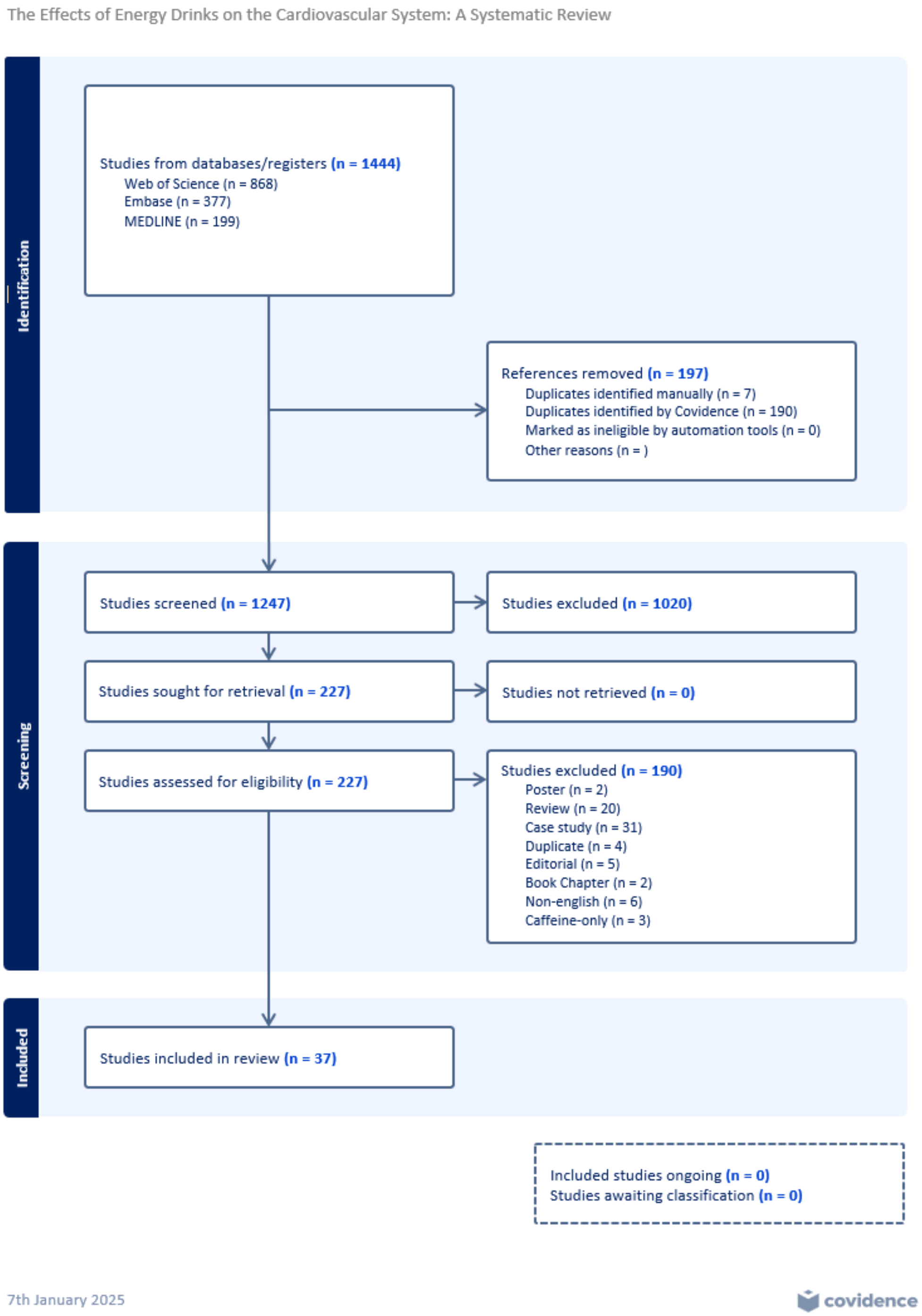

Fig 1. The figure above represents the systematic review methodology, including initial articles, abstract review, and full text review (with displayed exclusion criteria) to achieve the final 37 studies screened for review. Covidence (NIH, Bethesda, MD) populated into PRISMA and was used to achieve this methodology.

Systematic Review Data Extraction Template	
Covidence #	* Heart Rate (BPM): (Mean, Standard Deviation, P-Value)
Study ID	* Heart Rate (BPM): (Mean, Standard Deviation, P-Value)
Study Title	* Heart Rate Variability: (Mean, Standard Deviation, P-Value)
Lead Author	* Systolic Blood Pressure (mmHg): (Mean, Standard Deviation, P-Value)
Date Published	* Diastolic Blood Pressure (mmHg): (Mean, Standard Deviation, P-Value)
Country	* Mean Arterial Pressure (mmHg): (Mean, Standard Deviation, P-Value)
Aim of Study	* Cardiac Output (L/min): (Mean, Standard Deviation, P-Value)
Study design	* Stroke Volume (L): (Mean, Standard Deviation, P-Value)
Study Conclusions	* EKG Conducted (Yes/No)
Author Funding sources	* Specific ECG QT/QTc Changes: (Described)
Author Conflicts of Interest	* Other ECG Changes/Arrythmias: (Described)
Population Description	* Patient Reported Symptoms: (Described)
Inclusion criteria	**Quality Of Study Extraction:**
Exclusion criteria	Allocation concealment: (High/Low/Unsure)
Method of Recruitment of Participants	Blinding of Participants and Personnel: (High/Low/Unsure)
Total Number of Participants	Blinding of outcome assessment: (High/Low/Unsure)
Baseline Population Characteristics: (Mean Age, Race, Sex, BMI)	Incomplete outcome data: (High/Low/Unsure)
* Intervention and Comparisons: (Energy Drink Used, Caffeine Dosage Exercise (Y/N)	Selective reporting: (High/Low/Unsure)

* = Each intervention (Experimental/Control) had its own data reported

Table 1. Systematic Review Data Extraction Template: The figure above represents the systematic review criteria the authors gathered for the final 37 studies screened for review. The lighted shaded area represents data that was extracted from the Data Extraction Template via Covidence (NIH, Bethesda, MD). The Darker shaded area represents the quality of study endpoints from Covidence (NIH, Bethesda, MD) reviewed and confirmed by consensus via the authors on after data extraction

Extracted Studies Pertinent Data			
Title	Energy Drink Used Intervention 1	Total Number Of Participants	Caffeine Dosage Intervention 1
Acute Effects Of An Energy Drink On Myocardial Function Assessed By Conventional Echo-doppler Analysis And By Speckle Tracking Echocardiography On Young Healthy Subjects	Unknown	35	168 Ml/M2 (BSA, Gehan & George) Of An Energy Drink Containing Caffeine (0.03%), Taurine (0.4%), Glucuronolactone (0.24%), Glucose, And Other Ingredient)
Acute Effects Of Caffeinated Beverages On Electrocardiographic And Hemodynamic Parameters In Young Adults	Redbull	56	151.4mg
Acute Effects Of Commercial Energy Drink Consumption On Exercise Performance And Cardiovascular Safety: A Randomized, Double-blind, Placebo-controlled, Crossover Trial	Control	30	0
Acute Effects Of Energy Drink Consumption On Microvascular Reactivity In Young Male Volunteers At Rest: A Randomized Trial	Red Bull Brazil	32	80mg Caffiene
Acute Effects Of Energy Drink On Arterial Stiffness And Endothelial Function In Young Male Bodybuilders With Habitual Caffeine Consumption	Monster Energy Drink	45	X
Acute Effects Of Energy Drink On Hemodynamic And Electrophysiologic Parameters In Habitual And Non-habitual Caffeine Consumers	Redbull	48	151.36mg
Acute Effects Of Energy Drink On Ventricular Repolarization In Healthy Young Volunteers: A Prospective Study	Redbull	50	114 Mg
Acute Effects Of Red Bull Energy Drinks On Atrial Electromechanical Function In Healthy Young Adults	330ml Redbull	54	114mg
Adrenergic System Activation Mediates Changes In Cardiovascular And Psychomotoric Reactions In Young Individuals After Red Bull; Energy Drink Consumption	Redbull 500ml	38	Unknown
Cardio- And Cerebrovascular Responses To The Energy Drink Red Bull In Young Adults: A Randomized Cross-over Study	Redbull	25	114mg
Cardiovascular And Cerebrovascular Effects In Response To Red Bull Consumption Combined With Mental Stress	Redbull	20	114mg
Cardiovascular Hemodynamic Effects Of Red Bull R Energy Drink During Prolonged, Simulated, Monotonous Driving	Redbull	12	80 Mg Caffeine
Cardiovascular Responses To ENERGY Drinks In A Healthy Population During Exercise: The C-energy-x Study	Monster Energy Drink	23	80 Mg Per 8 Oz Serving
Cardiovascular Responses To Energy Drinks In A Healthy Population: The C-energy Study	Monster Energy Drink	14	Unknown
Cerebro- And Cardio-vascular Responses To Energy Drink In Young Adults: Is There A Gender Effect?	Red Bull	45	114
Consumption Of Energy Drinks And Assessment Of Blood Pressure Values Among Young Adults	Self-reported Consumption	309	Caffeine
Effect Of Energy Drink Consumption On Baroreceptor Sensitivity In Young Normal Weight And Overweight/Obese Males	Unknown	25	80 Mg Per Can, Consumed 5 Ml/Kg Of Can

Effect Of Energy Drink Dose On Exercise Capacity, Heart Rate Recovery And Heart Rate Variability After High-intensity Exercise	Weight-adjusted Caffeine	15	0
Effects Of Energy Drink Consumption On Corrected QT Interval And Heart Rate Variability In Young Obese Saudi Male University Students	Unknown	31	Unknown
Effects Of Energy Drinks On Blood Pressure, Heart Rate, And Electrocardiographic Parameters: An Experimental Study On Healthy Young Adults Reply	250ml Of Unkown Energy Drink	44	80mg
Effects Of Single And Multiple Energy Shots On Blood Pressure And Electrocardiographic Parameters	5 Hr Energy 2 Ounces	26	200
Electrocardiographic And Blood Pressure Effects Of Energy Drinks And Panax Ginseng In Healthy Volunteers: A Randomized Clinical Trial	Two 16 oz Containers Of A Popular Energy Drink, 800 mg Of P. Ginseng In 70 ml Of Cherry Syrup, 20 ml Of Lime Juice And 410 ml Of Carbonated Water	27	320 Mg
Energy Drink Effects On Hemodynamics And Endothelial Function In Young Adults	24 Oz Monster	44	240mg
Energy Drinks And Their Acute Effects On Arterial Stiffness In Healthy Children And Teenagers: A Randomized Trial	Unknown	27	3 Mg Caffeine Per Kilogram Of Body Weight Per Day
Energy Drinks And Their Acute Effects On Heart Rhythm And Electrocardiographic Time Intervals In Healthy Children And Teenagers: A Randomized Trial	Commercially Available Caffeinated ED	26	3 Mg Per Kg Of Bodyweight
Energy Drinks Decrease Left Ventricular Efficiency In Healthy Children And Teenagers: A Randomized Trial	Commercially Available	24	3 Mg/Kg
Energy Drinks Induce Acute Cardiovascular And Metabolic Changes Pointing To Potential Risks For Young Adults: A Randomized Controlled Trial	Redbull 750ml	38	240mg
Energy Drinks: Effects On Blood Pressure And Heart Rate In Children And Teenagers. A Randomized Trial	Commercially Available	27	Bodyweight-adjusted For Maximal Caffeine Consumption For Healthy Children (3 Mg Per Kg Of Bodyweight)
Energy Drinks: Effects On Pediatric 24-h Ambulatory Blood Pressure Monitoring. A Randomized Trial	Commercially Available Energy Drink	17	3 Mg Per Kg Of Bodyweight
Flying High?-Exploring The Effect Of Red Bull On Blood Pressure After Microsurgical Breast Reconstruction In A Randomized Controlled Trial	Red Bull	100	3 Doses Of 250 Ml Of Red Bull Beverage
Impact Of High Volume Energy Drink Consumption On Electrocardiographic And Blood Pressure Parameters: A Randomized Trial	"Drink A" 2 16oz Bottles	34	304-320mg
Low-calorie Energy Drink Improves Physiological Response To Exercise In Previously Sedentary Men: A Placebo-controlled Efficacy And Safety Study	Celsius	37	200 Mg
Non-linear Analysis Of Heart Rate Variability For Evaluating The Acute Effects Of Caffeinated Beverages In Young Adults	Redbull Energy	48	151.4mg
Novel Energy Drink Improves Cognitive Function And Mood, Without Influencing Myocardial Oxygen Demand Or Ventricular Repolarization In Adult Gamers: A Randomized, Double-blind, Placebo-controlled, Crossover Trial	C4s	45	200mg
Synergistic Effect Of Energy Drinks And Overweight/Obesity On Cardiac Autonomic Testing Using The Valsalva Maneuver In University Students	Red Bull	50	80 Mg

The Blood Pressure-elevating Effect Of Red Bull Energy Drink Is Mimicked By Caffeine But Through Different Hemodynamic Pathways	Red Bull	8	32 Per 100 Ml
The Effect Of Acute Consumption Of Energy Drinks On Blood Pressure, Heart Rate And Blood Glucose In The Group Of Young Adults	Most Popular Energy Drink At Local Store	68	80 Mg Of Caffeine

Table 2. Extracted Data: Table containing studies extracted, energy drink tested, total study population, and amount of caffeine used for the study design. All studies were completed later than 2010.
